# Social determinants of mental health care systems: intensive community based Care in the Veterans Health Administration

**DOI:** 10.1186/s12889-020-09402-0

**Published:** 2020-08-28

**Authors:** Ish P. Bhalla, Elina A. Stefanovics, Robert A. Rosenheck

**Affiliations:** 1 Yale University Department of Psychiatry, 950 Campbell Ave, Building 35, West Haven, CT 06516 USA; 2 University of California, Los Angeles National Clinician Scholars Program, 1100 Glendon Ave, Suite 900, Los Angeles, CA 90024 USA; 3 Veterans Affairs New England Mental Illness Research Education, and Clinical Center, West Haven, USA; 4 Yale University School of Public Health, 950 Campbell Ave, Building 35, West Haven, CT 06516 USA

## Abstract

**Background:**

Since deinstitutionalization in the 1950s–1970s, public mental health care has changed its focus from asylums to general hospitals, outpatient clinics and specialized community-based programs addressing both clinical and social determinants of mental health. Analysis of the place of community-based programs within a comprehensive health system such as the Veterans Health Administration (VHA) may illuminate the role of social forces in shaping contemporary public mental health systems.

**Methods:**

National VHA administrative data were used to compare veterans who exclusively received outpatient clinic care to those receiving four types of specialized community-based services, addressing: 1) functional disabilities from severe mental illness (SMI), 2) justice system involvement, 3) homelessness, and 4) vocational rehabilitation. Bivariate comparisons and multinomial logistic regression analyses compared groups on demographics, diagnoses, service use, and psychiatric prescription fills.

**Results:**

An hierarchical classification of 1,386,487 Veterans who received specialty mental health services from VHA in Fiscal Year 2012, showed 1,134,977 (81.8%) were seen exclusively in outpatient clinics; 27,931 (2.0%) received intensive SMI-related services; 42,985 (3.1%) criminal justice services; 160,273 (11.6%) specialized homelessness services; and 20,921 (1.5%) vocational services. Compared to those seen only in clinics, veterans in the four community treatment groups were more likely to be black, diagnosed with HIV and hepatitis, had more numerous substance use diagnoses and made far more extensive use of mental health outpatient and inpatient care.

**Conclusions:**

Almost one-fifth of VHA mental health patients receive community-based services prominently addressing major social determinants of health and multimorbid substance use disorders.

## Background

Care for people with psychiatric disorders has undergone extraordinary changes in the past 70 years from a focus on asylum care to a “de facto” system of diverse, largely non-institutional services [1–3]. A distinctive feature is the provision of community-based often intensive services for the most vulnerable, those long thought to be the most inadequately served [4]. In 1950, care for people with SMI, provided in over 500,000 state mental hospital beds [5], and was a target of public scorn [6]. By 1970, the majority of these beds had been closed and acute care was provided primarily in general hospitals, with longer term institutional care in nursing and board and care homes [7], and outpatient care in public clinics bolstered by newly developed antipsychotic and other psychiatric medications. By 1980, a substantial academic literature had developed decrying the failures of de-institutionalization and the neglect of people with the greatest needs [8]. Researchers showed that Assertive Community Treatment (ACT) [9] and other forms of intensive community-based care could provide humane services in non-institutional settings [10] at little or no additional cost [11]. Here too, critics claimed programs were under-funded [12]. Specialized psychiatric rehabilitation services were also developed to restore community adaptation and productive functioning [13–15], but these services were also believed to be of limited availability [16, 17].

In the 1980s an unanticipated crisis of homelessness emerged. Initially viewed as a failure of deinstitutionalization because many homeless adults had SMI [18], it was eventually recognized to be more a consequence of the loss of affordable housing and the decline in public income support [19, 20] - a one-two punch that fell hard on people with SMI and addictions [21]. This highly visible subgroup of homeless adults was recognized to need income, housing and specialized community outreach services as well as psychiatric care.

In an apparent rebound of institutionalism, the criminal the justice system exploded, in large part due to harsh new drug laws, and became an unwanted new asylum for people with psychiatric disorders representing, to many, a de facto criminalization of mental illness [22–24]. In response, diversion programs were designed to create a channel from the criminal justice system to mental health services [25].

The current system of community-based care for people with SMI thus developed in response both biomedical innovations and what has increasingly been referred to as social determinants of mental health [26] (i.e. social determinants of mental illness) [27–29]. The result has been a non-institutional system composed of two broad components: a standard clinic-based component backed by a limited hospital capacity, that serves the majority of patients, providing medications and behavioral therapies; and a second, outwardly facing, community focused component providing more resource intensive services to patients most impacted by “social determinants” and in need of specialized in vivo care. These community-based services were initially conceptualized as replacing care previously provided by state hospitals, but, as suggested above, they also emerged in response to a broad array of social and economic developments.

The Veterans Health Administration (VHA) of the United States Department of Veterans Affairs (VA) is a nationally integrated health system with a specific mission to provide comprehensive healthcare to eligible veterans of military service, i.e. those discharged from active military duty under conditions other than dishonorable with special priorities for those with service-related disabilities, possible post-traumatic stress disorder, or exposed to military sexual trauma [30]. VA also offers income benefits to veterans who incurred adverse health effects during military service and other rehabilitative, educational and home mortgage benefits.

In many ways VHA mental health care has followed the same evolution as outlined above in other public mental health systems, with extensive bed closures, expansion of outpatient services, and use of the same biomedical innovations. VHA also responded to the same social phenomena with specialized community-based services although these services are only available to veterans formally enrolled in VHA, and in many cases such services may be more available in VHA that elsewhere in other US mental health systems [31]. The VHA is distinctive in that its electronic health records system comprehensively documents sociodemographic characteristics and clinical diagnoses as well as service use and prescribed medications of those it serves. VHA data thus offer a unique opportunity to empirically examine place of intensive community-based mental health services in a twenty-first century system of care and the distinctive characteristics of the veterans it cares for.

This study uses national VHA data on 1.3 million veterans who received specialized mental health services from VHA in FY 2012, 240,000 (18%) of whom received often intensive community-based services that can be classified in four types: 1) ACT-like intensive case management and recovery-oriented day program services for veterans disabled by serious mental illness (SMI) [32–34]; 2) outreach service to veterans involved in the criminal justice system [35–38]; 3) outreach and housing services for homeless veterans [39–43], and 4) rehabilitation and community-based employment services [44–46]. The primary goal of the criminal justice programs was outreach and linkage to standard VHA outpatient services. We included these programs in this study because of they represent a community-based service related to social problems as well as medical/psychiatric ones. In fact, historically, these programs were an outgrowth of the development of community-based programs for homeless veterans, many of whom have criminal justice problems. While these programs have been studied individually, no studies have taken a broad view of the development of community-based programs within the VHA mental health care system and examined the characteristics of veterans served by these programs together in a single analysis that examined their relative size and compared their participants to veterans served by standard outpatient clinics.

In this study veterans receiving these four specialized community-based services are compared to those who received only clinic-based services on socio-demographic and diagnostic characteristics and on patterns of mental health and medical service use. While a proportion of veterans were served by multiple programs, our primary intention was to describe how these programs can be understood together as a community-based response to diverse social determinants of health. We further compare the veterans they serve to those treated in standard outpatient mental health clinics. Intensive community-based services for veterans, particularly case management services, were originally designed to provide psychiatric treatment to people with SMI. Our central observation is that the VHA responded to social determinants of veteran health in the community and designed programs to serve these veterans in vivo where their complex social issues arose. There have been many efforts in recent years to develop a comprehensive definition of social determinants of health [47, 48] and all have involved multiple, complex dimensions of functioning, general well-being, and social disadvantage. We take a less ambitious, more incrementalist approach to this issue examining the way a few, highly visible, social problems spurred the development of a new array of VHA programs. In this process VHA reached out to veterans with both distinctive social problems and complex mental health difficulties characterized by behavioral multimorbidities that could be addressed by one or another, or sometimes by more than one of these programs.

There has been particular interest in recent years in multi-morbidity, the co-occurrence of mutually exacerbating psychiatric, substance use and medical disorders which are responsible for severe functional impairments and place extensive demands on health care systems [49, 50]. We sought to pay simultaneous attention to both key social determinants of health that impacted Veterans, and their clinical multi-morbidity in an examination of factors that might illuminate the place of intensive community-based programs in contemporary mental health service delivery.

## Methods

### Sample

Using national VHA data from FY 2012, a total of 1,386,487 veterans were identified who had used specialty mental health care. These veterans were classified into groups by the types of services they received. Community based services include those predominantly delivered outside the offices of the health care system to directly address social risks to health such as homelessness, incarceration, poor social functioning, poverty, and lack of employment skills. These services are not always provided outside of health system facilities, but they are all heavily focused on practical skills, supports for community living, and addressing individual social as well as medical circumstances. Since some veterans receive services from multiple programs, we classified them hierarchically, for analytic purposes, in mutually exclusive categories, including first, the most intensive long-term programs for SMI, followed by the two outreach programs addressing veterans involved in the criminal justice system and/or who were homeless, and then psychiatric rehabilitation programs, often provided as an ancillary to other clinical services. The remaining group was veterans seen only in office-based outpatient clinics. Thus while some veterans were treated in more than one community program (21.3% of those seen in any community program), they were only included in one community treatment group in our analytic classification and most veterans seen in intensive community based programs (62.2%) were also seen in clinic settings.

### Measures

Measures, obtained from a pre-constructed dataset from the Northeast Program Evaluation Center, documented sociodemographic variables including age, sex, race, geographic residence (urban or rural), national region, income, VA pension status, service-connection disability status (VA income support programs), and homelessness in the past year (identified through use of specialized homeless services and the V60 ICD9 code). Geographic measures were obtained through zip codes using the Rural-Urban Commuting Area classification [51].

Medical diagnoses were selected based on those included in the Charlson comorbidity index, an aggregate measure of comorbidity that predicts 1-year mortality using a weighted sum of medical comorbidities [52]. In addition to the Charlson index itself, medical diagnoses known to be associated with mental illness and substance use were included, such as hepatic disease, human immunodeficiency virus infection (HIV) and pain diagnoses, using an array of codes described elsewhere [53].

Psychiatric diagnoses included schizophrenia, bipolar disorder, major depressive (ICD-9.

296.2–296.39) and other depressive disorders (ICD-9300.4x, 296.9x, 301.10–301.19, 311.x), posttraumatic stress disorder (ICD-9309.81), anxiety disorders (ICD-9300.xx excluding 300.4), and personality disorders (ICD-9301.9). In addition, 7 drug use disorders were included in the analysis: opiate (ICD-9304.0x or 305.5), cannabis (ICD-9304.3x or 305.2), cocaine (ICD-9304.2x or 305.6), barbiturates (ICD-9304.1x), amphetamines (ICD-9304.4x or 305.7), and hallucinogens (ICD-9304.5x or 305.3).

As a measure of multi-morbidity, summary variables were created as a count of the number of medical diagnoses, psychiatric diagnoses, and substance use diagnoses, and the total number of psychiatric and substance use diagnoses.

VHA outpatient health service utilization was derived from clinic stop codes (specific codes are available upon request) representing general psychiatric care, substance use specialty care, primary care, emergency department visits, and each of the four types of community psychiatric care.

Psychotropic medication fills were classified as antipsychotics, antidepressants, anxiolytic/sedative/hypnotics, stimulants, anticonvulsants/mood stabilizers and lithium.

### Analysis

Bivariate analyses were used to compare veterans treated only in mental health clinics to those who received services from each of the four hierarchically classified types of intensive community based services.

Because the subgroups examined in involve many tens of thousands of Veterans, small group differences with little clinical importance would likely be statistically significant. We thus relied on effect sizes to identify substantial differences between groups. Cohen’s *d* was calculated for continuous variables (the difference in means between groups divided by their pooled standard deviation); and risk ratios for dichotomous variables representing proportions. A cutoff value of > 0.20 or < − 0.20 was used as a threshold for at least a small difference in Cohen’s *d* [54] and risk ratios of > 1.5 or < 0.67 for dichotomous variables.

Multinomial logistic regression analysis was then used to identify the set of measures that independently differentiated veterans who had been treated in each of the four sub-specialty community health programs from those treated in mental health clinics only. Variables included in the multivariable analyses were those we had previously identified as being substantially different between the groups based effect size differences in bivariate comparisons.

All analyses were conducted using SAS statistical software (version 9.2; SAS Institute Inc., Cary, NC).

## Results

Among the total of 1,386,487 veterans who had received specialty mental health services, 252,110 (18.2%) received specialized intensive community-based services. In our unduplicated hierarchical classification 27,931 (2.0%) were classified in the intensive SMI services group; 42,985 (3.1%) in the criminal justice outreach group; 160,273 (11.6%) in homelessness services; and 20,921 (1.5%) in vocational services.

Bivariate analysis showed that veterans treated in criminal justice, homeless and vocational programs were substantially younger than those seen exclusively in outpatient mental health clinics. Veterans in the employment program group but not in the SMI program group had lower incomes (see comparisons using Cohen’s d in the right hand columns of Table 1). Veterans seen in each of the four community-based programs were substantially more likely to be black, and less likely to be from isolated rural areas.

**Table 1:** Bivariate comparison of demographic characteristics of veterans in community care treatment and mental health clinics only^a^

	Mental Health Clinic (1)		Vocational (2)		Homelessness (3)		Criminal Justice (4)		SMI Services (5)		2 vs 1	3 vs 1	4 vs 1	5 vs 1
N=	**1,134,377**		**20,921**		**160,273**		**42,985**		**27,931**		**Effect Size**			
Demographics	**Mean**	**SD**	**Mean**	**SD**	**Mean**	**SD**	**Mean**	**SD**	**Mean**	**SD**	**Cohen d**			
Age (years)	55.22	15.45	48.69	12.30	52.15	12.01	52.18	15.64	54.02	11.96	−0.43	− 0.20	− 0.20	−0.08
Income ($)	25,339.03	42,872.53	15,658.00	22,245.30	12,602.47	22,973.72	19,912.26	42,170.73	20,693.33	23,364.20	−0.24	−0.31	−0.13	− 0.11
	**%**	**N**	**%**	**N**	**%**	**N**	**%**	**N**	**%**	**N**	**Risk Ratio**			
Male	90.3	1,024,217	89.7	18,766	90.5	145,034	94.5	40,611	88.5	24,714	0.993	1.002	1.046	0.980
Race														
White	77.5	879,022	62.0	12,968	52.8	84,636	68.1	29,281	65.0	18,149	0.800	0.681	0.879	0.839
Black	16.2	183,291	31.2	6522	39.1	62,738	25.0	10,766	28.5	7957	1.929	2.423	1.550	1.763
Hispanic	17.0	192,678	14.7	3079	12.6	20,138	11.8	5059	10.0	2789	0.867	0.740	0.693	0.588
Mixed	2.1	23,692	2.5	516	2.7	4356	2.1	910	3.2	898	1.180	1.301	1.014	1.540
Other	1.6	18,251	1.2	247	1.1	1716	1.4	606	1.7	473	0.733	0.665	0.876	1.054
Residence type														
Urban area residence	68.8	780,219	78.9	16,499	85.0	136,183	76.5	32,877	81.4	22,737	1.147	1.235	1.112	1.184
Large rural area residence	11.5	130,283	9.5%	1979	6.7%	10,688	9.4%	4057	8.3%	2330	0.824	0.581	0.822	0.726
Small rural area residence	8.9	100,520	5.1%	1075	4.0%	6372	6.7%	2886	4.6%	1297	0.580	0.449	0.758	0.524
Isolated rural area residence	6.7	75,455	3.6%	756	2.6%	4144	5.2%	2233	2.7%	750	0.543	0.389	0.781	0.404
OEF/OIF era	17.6	200,092	19.5	4085	9.9	15,798	17.9	7715	8.8	2453	1.107	0.559	1.018	0.498
Service connected 50% or more	39.1	444,072	20.2	4227	15.0	24,062	20.5	8830	43.2	12,074	0.516	0.384	0.525	1.104
Service connected < 50%	18.9	214,572	19.7	4117	17.0	27,195	16.1	6907	11.7	3264	1.040	0.897	0.849	0.618
Homeless during the year	1.4	15,932	10.7	2235	86.2	138,219	30.6	13,169	28.3	7894	7.606	61.404	21.813	20.123

^a^Non-substantial differences involving less than 5% of the sample are not shown. Data available on request

Veterans in the criminal justice, homeless and vocational program groups were all less likely to have a service-connected disability rating of 50% or more than those seen in clinics. There were few differences on the Charlson index of medical co-morbidity although those in vocational program group had a lower index of medical problems than those seen in clinics, and veterans treated in each of the four community program groups had greater risks of HIV and hepatic diagnoses.

Most dramatic were the substantially 2–3 times greater numbers with any drug or alcohol abuse or dependence diagnoses in all four community-based program groups as compared to the clinic group, with Cohen’s *d*’s of greater than 0.5 for the total number of such diagnoses and risk ratios for each specific drug and alcohol use diagnosis greater than 2.0 (Table 2).

**Table 2:** Bivariate comparison of diagnoses of veterans in community care treatment and mental health clinics only^a^

	Mental Health Clinic (1)		Vocational (2)		Homelessness (3)		Criminal Justice (4)		SMI Services (5)		2 vs 1	3 vs 1	4 vs 1	5 vs 1
**N=**	**1,134,377**		**20,921**		**160,273**		**42,985**		**27,931**		**Effect Size**			
	**Mean**	**SD**	**Mean**	**SD**	**Mean**	**SD**	**Mean**	**SD**	**Mean**	**SD**	**Cohen d**			
Multimorbidity														
Number of psychiatric diagnoses	1.79	1.11	1.87	1.42	1.60	1.49	1.43	1.53	2.62	1.483	0.067	−0.151	−0.294	0.688
Number of SUD diagnoses	0.27	0.62	0.70	1.04	0.87	1.16	0.75	1.13	0.80	1.130	0.571	0.797	0.643	0.708
Number of psychiatric and SUD diagnoses	2.05	1.33	2.56	1.91	2.47	2.18	2.18	2.27	3.42	2.068	0.332	0.272	0.084	0.885
Charlson medical severity diagnosis index	2.81	2.44	1.96	1.99	2.32	2.16	2.45	2.53	2.80	2.185	−0.347	−0.199	−0.144	− 0.001
Medical diagnosis	**%**	**N**	**%**	**N**	**%**	**N**	**%**	**N**	**%**	**N**	**Risk Ratio**			
Insomnia	8.2	92,536	8.3	1736	6.6	10,592	6.3	2709	6.6	1830	1.017	0.810	0.773	0.803
Congestive heart failure	48.7	552,483	47.4	9911	48.6	77,896	46.0	19,793	61.5	17,184	0.973	0.998	0.945	1.263
Chronic obstructive pulmonary	14.8	167,951	12.5	2623	14.7	23,540	14.3	6165	19.4	5412	0.847	0.992	0.969	1.309
Diabetes mellitus	23.3	264,173	16.7	3487	17.0	27,240	16.9	7283	27.0	7535	0.716	0.730	0.728	1.158
Cancer	7.2	81,768	3.9	822	5.1	8208	6.2	2677	5.8	1619	0.545	0.710	0.864	0.804
Human immunodeficiency virus	0.5	6226	1.4	287	1.5	2420	0.9	369	1.0	275	2.499	2.751	1.564	1.794
Headache	9.5	107,605	12.0	2514	8.8	14,084	8.4	3610	10.5	2926	1.267	0.926	0.885	1.104
Any pain	56.2	637,256	63.0	13,186	58.4	93,525	52.7	22,673	61.1	17,054	1.122	1.039	0.939	1.087
Psychiatric Diagnosis														
Any psychiatric disorder	92.2	1,045,820	85.9	17,972	78.2	125,382	68.4	29,403	99.4	27,757	0.932	0.849	0.742	1.078
Schizophrenia	4.8	54,401	6.5	1367	6.3	10,148	3.9	1662	47.8	13,360	1.362	1.320	0.806	9.974
Other psychotic disorder	2.5	28,651	3.8	801	4.4	7094	3.6	1529	13.9	3878	1.516	1.752	1.408	5.497
Bipolar disorder	6.9	78,011	11.2	2333	9.0	14,406	7.5	3236	22.9	6395	1.622	1.307	1.095	3.329
Major depressive disorder	21.1	239,616	22.9	4799	17.1	27,390	13.9	5987	26.9	7512	1.086	0.809	0.659	1.273
Other depressive disorders (e.g., dysthymia)	43.7	495,951	44.9	9399	39.5	63,253	33.2	14,263	41.5	11,589	1.028	0.903	0.759	0.949
PTSD	41.8	473,699	30.2	6323	24.7	39,653	25.4	10,937	34.8	9717	0.724	0.592	0.609	0.833
Anxiety disorder	26.1	296,466	26.0	5447	19.8	31,672	18.8	8070	25.9	7238	0.996	0.756	0.718	0.992
Adjustment disorder	10.2	115,639	13.4	2805	13.5	21,685	12.2	5245	10.0	2787	1.315	1.327	1.197	0.979
Personality disorder	2.7	30,676	6.1	1283	5.9	9442	5.5	2375	11.5	3226	2.268	2.179	2.043	4.271
Other psychiatric diagnosis	18.8	213,714	21.5	4506	20.2	32,454	19.2	8269	26.4	7367	1.143	1.075	1.021	1.400
Dual diagnosis	19.0	215,937	39.2	8191	44.8	71,813	38.4	16,527	43.9	12,251	2.057	2.354	2.020	2.304
Substance Use Disorder														
Any substance use disorder	9.9	112,789	28.3	5917	35.5	56,838	29.5	12,662	33.5	9347	2.845	3.567	2.963	3.366
Alcohol	13.8	156,291	29.5	6173	33.9	54,290	30.1	12,938	31.3	8732	2.142	2.459	2.185	2.269
Cannabis	3.8	42,884	9.8	2057	11.9	19,030	10.1	4348	12.1	3378	2.601	3.141	2.676	3.199
Cocaine	2.5	28,593	12.3	2565	17.7	28,334	13.1	5610	15.0	4201	4.864	7.014	5.178	5.967
Opioid	3.9	44,190	13.0	2716	17.2	27,577	15.8	6797	15.9	4436	3.333	4.417	4.059	4.077
Sedative/hypnotic	0.5	5127	1.2	241	1.4	2278	1.5	625	1.5	431	2.549	3.145	3.217	3.414
Amphetamine	0.5	5969	1.7	352	2.6	4246	2.9	1244	2.2	627	3.198	5.035	5.500	4.266

^a^Non-substantial differences involving less than 5% of the sample are not shown. Data available on request

Numbers of non-substance use psychiatric diagnoses were substantially greater in the SMI program group with Cohen’s *d* of .69 and fewer in the criminal justice programs with Cohen’s *d* of −.29. Individual diagnoses most strongly associated with the SMI programs included schizophrenia, and bipolar disorder. While proportions of veterans diagnosed with personality disorder were greater in all four community program groups than in outpatient clinics, they were 4 times more common in the SMI programs even though personality disorder is not considered a serious mental illness (Table 2).

Veterans in each of the four community program groups had far more psychiatric and substance use outpatient visits than those seen in outpatient mental health clinics (Table 3), and participants in the SMI and vocational programs had more general psychiatry visits over and above the visits to specialized community service programs themselves, and three times as many total mental health outpatient contacts (totaling 51.63/year) overall. Veterans in all community program groups were more likely to have been hospitalized for psychiatric treatment compared to those seen in mental health clinics alone. There were no substantial differences in primary care or medical specialty visits although veterans seen in the criminal justice and SMI programs were more likely to have had medical hospitalizations (Table 3).

**Table 3:** Bivariate comparison of service utilization and psychotropic prescription fills among veterans in community care treatment and mental health clinics only

	Mental Health Clinic (1)		Vocational (2)		Homelessness (3)		Criminal Justice (4)		SMI Services (5)		2 vs 1	3 vs 1	4 vs 1	5 vs 1
**N=**	**1,134,377**		**20,921**		**160,273**		**42,985**		**27,931**		**Effect Size**			
	**Mean**	**SD**	**Mean**	**SD**	**Mean**	**SD**	**Mean**	**SD**	**Mean**	**SD**	**Cohen d**			
General psychiatry visits	5.81	9.27	15.96	22.89	16.51	24.43	11.83	22.54	67.37	74.42	0.602	0.634	0.357	3.650
General psychiatry visits excluding community-based programs	5.81	9.27	10.60	20.01	8.04	19.65	6.00	16.69	15.98	28.61	0.389	0.181	0.016	0.826
Substance abuse clinic visits	0.90	7.91	7.76	25.47	8.08	26.01	8.31	24.94	7.17	25.34	0.515	0.539	0.556	0.471
Medical surgical visits	9.60	11.12	10.66	11.40	10.07	11.76	9.54	12.27	13.45	14.53	0.092	0.040	−0.006	0.334
Primary care visits	3.43	3.50	4.02	3.86	3.93	4.24	3.27	3.96	4.71	5.08	0.156	0.134	−0.044	0.344
Specialty medical clinic visits	6.17	9.35	6.64	9.40	6.14	9.52	6.26	10.16	8.74	12.12	0.049	−0.003	0.010	0.267
Emergency room visits	0.60	1.58	1.06	2.32	1.37	2.97	1.22	2.67	1.87	4.13	0.232	0.388	0.313	0.644
	**%**	**N**	**%**	**N**	**%**	**N**	**%**	**N**	**%**	**N**	**Risk Ratio**			
Any mental health inpatient treatment	2.8	32,134	8.0	1674	10.3	16,581	9.4	4029	25.2	7037	2.825	3.652	3.309	8.894
Any medical surgical inpatient treatment	7.9	90,143	8.7	1814	11.6	18,563	12.0	5154	14.3	4007	1.091	1.458	1.509	1.805
Psychotropic medication prescriptions	**Mean**	**SD**	**Mean**	**SD**	**Mean**	**SD**	**Mean**	**SD**	**Mean**	**SD**	**Cohen d**			
Antidepressant prescriptions	5.67	9.79	6.45	16.22	5.84	13.63	5.26	13.52	12.37	25.31	0.069	0.015	−0.037	0.595
Antipsychotic prescriptions	1.76	8.09	2.65	11.73	2.53	10.53	2.06	9.29	16.41	35.24	0.089	0.077	0.030	1.467
Anxiolytic/sedative/hypnotic prescriptions	2.90	5.64	2.29	6.57	1.92	5.71	1.78	5.33	4.77	9.73	−0.104	−0.167	−0.192	0.318
Stimulant prescriptions	0.16	1.28	0.20	3.52	0.10	1.74	0.10	1.38	0.13	1.32	0.024	−0.042	−0.042	−0.024
Anticonvulsant/Mood Stabilizer Prescriptions	1.55	6.09	2.08	8.64	2.19	9.04	2.04	9.04	7.12	21.19	0.072	0.087	0.067	0.756
Lithium prescriptions	0.13	1.65	0.25	2.51	0.21	2.51	0.18	2.18	1.47	9.95	0.052	0.033	0.022	0.577
All Psychotropics	13.03	21.03	14.74	33.21	14.40	30.33	13.85	30.64	43.05	70.05	0.068	0.054	0.033	1.189
Opiate Prescriptions	7.51	7.50	6.72	7.70	7.20	8.32	6.81	8.61	7.49	9.38	−0.101	−0.040	−0.090	−0.002
Intensive community treatment programs	**Mean**	**SD**	**Mean**	**SD**	**Mean**	**SD**	**Mean**	**SD**	**Mean**	**SD**	**Cohen d**			
SMI Services	0.00	0.00	0.00	0.00	0.00	0.00	0.00	0.00	46.58	67.38	n/a	n/a	n/a	n/a
Criminal justice outreach, jail diversion visits	0.00	0.00	0.00	0.00	0.00	0.00	2.45	4.27	0.19	1.64	n/a	n/a	n/a	n/a
Homelessness visits	0.00	0.00	0.00	0.00	7.49	10.95	2.70	8.45	3.30	10.08	n/a	n/a	n/a	n/a
Vocational rehabilitation visits	0.00	0.00	5.36	9.63	1.54	5.93	1.03	5.05	1.56	6.08	n/a	n/a	n/a	n/a
Total intensive community treatment visits	0.00	0.00	5.36	9.63	9.04	13.41	6.18	12.46	51.63	68.38	n/a	n/a	n/a	n/a

Veterans served by the SMI program group also had three times as many psychotropic prescription fills as those only seen in outpatient clinics, but substantial differences in psychotropic prescription fills were not seen in association with other community-based programs. Veterans who received SMI services filled far more antipsychotic prescriptions. There were no substantial differences in numbers of prescriptions for opiates (Table 3).

Multinomial logistic regression showed that, independent of other factors, veterans in the homeless and vocational program groups had lower incomes and were less likely to have a service-connected disability status of greater than 50% than those in the mental health clinic group. Veterans serviced by criminal justice programs had a lower total number of non-substance use psychiatric diagnoses (psychiatric multi-morbidity) whereas those in SMI programs had a substantially higher number of such diagnoses. Perhaps the most dramatic independent association was that Veterans in each of the four community program groups were diagnosed with more numerous substance use disorders than those in the clinic group (Table 4).

**Table 4:** Multinomial logistic regression with multimorbidity characteristics, comparing veterans treated in community psychiatry care with those in mental health

	Vocational		Homeless		Criminal Justice		SMI Services	
	OR	Standardized Regression coefficient	OR	Standardized Regression coefficient	OR	Standardized Regression coefficient	OR	Standardized Regression coefficient
Demographics								
Age	0.977	−0.191**	0.993	−0.0574**	0.991	−0.075**	1.002	0.015**
Income	1.000	−0.200**	1.000	−0.413**	1.000	−0.040**	1.000	−0.062**
Black race	2.040	0.156**	2.570	0.206**	1.422	0.077**	1.799	0.128**
Small rural area residence	0.638	−0.068**	0.509	−0.102**	0.836	−0.027**	0.513	−0.100**
Isolated rural area residence	0.635	−0.059**	0.478	−0.097**	0.896	−0.015**	0.406	−0.118**
Pension	1.142	0.014*	1.614	0.050**	1.018	0.002**	2.480	0.095**
Service connected 50% or more	0.466	−0.202**	0.394	−0.246**	0.493	−0.187**	1.279	0.065**
Diagnoses								
Connective tissue disease	0.822	−0.011	0.772	−0.014**	1.018	0.001**	0.643	−0.024**
Hepatic disease	1.207	0.021**	1.255	0.025**	1.014	0.002**	1.070	0.0070
Human immunodeficiency virus	1.480	0.018**	1.268	0.011**	1.011	0.001**	1.075	0.0030
Dementia	0.233	−0.094**	0.304	−0.077**	0.868	−0.009**	0.709	−0.022**
Multi-morbidity								
Number of psychiatric diagnoses	1.004	0.003	0.840	−0.115**	0.714	−0.222**	1.482	0.260**
Number of substance use disorder diagnoses	1.666	0.217**	1.953	0.285**	2.025	0.300**	1.663	0.217**
Service Use								
General psychiatry visits excluding community-based programs	1.019	0.130**	1.015	0.106**	1.009	0.062**	1.018	0.126**
Substance abuse clinic visits	1.018	0.132**	1.016	0.118**	1.019	0.141**	1.013	0.097**
Medical surgical clinic visits	1.012	0.076**	1.006	0.036**	1.008	0.048**	1.007	0.047**
Emergency room visits	1.065	0.070**	1.105	0.111**	1.107	0.113**	1.073	0.078**
Any mental health inpatient treatment	1.446	0.043**	2.138	0.089**	2.516	0.108**	3.267	0.139**
Any medical surgical inpatient treatment	0.850	−0.026**	1.081	0.013**	1.270	0.038**	0.899	−0.017**
All psychotropic medications	1.001	0.017*	1.000	−0.004	0.998	−0.033**	1.007	0.100**

*p < .01, **p < .0001

## Discussion

This study used national VHA data to compare the proportions and characteristics of the 82% of veterans treated exclusively in traditional mental health outpatient clinics to the 18% of veterans treated in VHA’s four major specialized community-based programs. Veterans treated in all four types of community programs were distinguished most strikingly, by being diagnosed with 2–3 times more numerous multimorbid substance use disorders, were more likely to have HIV and hepatic disease, to be from urban areas, of black race and also, as expected, had 3–13 times more mental health outpatient visits, most of which were in community-based programs themselves. Veterans served in the specialized programs for SMI veterans (only 2% of the total) were much more likely than clinic patients to be diagnosed with psychotic disorders, to manifest psychiatric multimorbidity and personality disorders and had 13 times more total visits, receiving over three times as many prescriptions for psychotropic medications.

In the decades after the closure of public psychiatric hospitals in the 1950s–70s, public mental health systems faced the question of how to address the broad needs of: 1) SMI patients who formerly would have been institutionalized, as well as the needs of emerging populations of 2) homeless people with mental illnesses; 3) criminal justice involved adults with mental health disorders; 4) veterans seeking rehabilitation/employment along with 5) a much larger group of people newly seeking effective care for less severe problems. The intensive community programs included in this study all originated in a push by VHA to develop care beyond the clinic in the community, primarily to better address the clinical challenges of SMI. At their inception, these programs were at times organizationally integrated [55] or co-located [56] together and with primary care services. While there has been extensive documentation of the reduction in long term State and VHA psychiatric hospital beds [5, 54, 55], and many studies of the growth of outpatient mental health treatment generally [56–58]; we know of no previous system-wide studies of the place of community-based services in any public mental health system nor of characteristics of people who use these services as compared to people served by standard outpatient clinics.

On the one hand, available studies have examined mental health service delivery in the US as a whole and have shown that “the system” faces major challenges with respect to the treatment engagement of people with serious mentally illness [57]. On the other, the National Comorbidity Survey (NCS) and NCS Replication show that between 1990 and 2003 basic treatment rates for people with mental illness increased significantly while the overall rates of mental illness did not change [58] although many remained underserved [59]. Additionally, among people with SMI, rates of any mental health treatment increased from 24.3 to 40.5% [58]. Furthermore, data from the Healthcare for Communities Survey showed an increase in mental health specialty treatment for people with SMI from 39% in 1997 to 51% in 2001 with an even larger increase (from 47 to 76%) for the subgroup who perceived a need for treatment [60]. These studies, however, did not examine community-based services, specifically, and most studies have focused on people with mild to moderate mental illness. For example, studies of the National Ambulatory Medical Care Survey found that treatment for depression tripled between 1987 and 1997 [61], and that most antidepressants are prescribed by primary care providers [62, 63].

Local studies based on Medicaid data do show that community programs continued to provide ACT and ACT-like services to the most seriously mentally ill and functionally impaired adults [64], though one recent survey suggested that less than 20% of non-VA community mental health facilities offer ACT [65] and even fewer offer other community services such as peer support, employment, and housing services [66]. While most research has focused on *either* people who use less intensive services (i.e. from standard mental health outpatient clinics) or specific community-based treatments like ACT or supported housing, no study to our knowledge has addressed the broad array of clinic *and* intensive community-based services offered together in a national system or even in one community. The present study, based on VHA data showed intensive community-based service are provided to 18% of those receiving any specialty mental health services especially to those with multiple substance use disorders, severe mental illness, criminal justice involvement, and/or homelessness. A previous study of VHA care showed that considering all patients with psychiatric diagnoses, one-third receive no specialty mental health treatment at all and receive care for mental disorders exclusively in primary or specialty care clinic settings [67]. That study and this one taken together, thus appear to be unique in mapping the major components of VHA mental health care, a comprehensive mental health system in which most patients receive care in standard outpatient mental health and primary care clinics but distinct subgroups receive intensive community-focused care largely shaped by social determinants and SUD-related multimorbidity.

In view of this perspective, it is notable that several recent reviews have emphasized the unique role of mental health services in addressing social determinants of health as well as individual biomedical conditions [26, 27, 68]. The portrait of community-based care in VHA presented here illustrates the way mental health systems have been shaped by a few major social determinants of health, an approach that differs from the more comprehensive approach taken by others [47, 48]. As clinicians, Sheilds-Zeeman described two types of intervention which are referred to as “social risk–informed” care and “social risk-targeted care.” Social risk–*informed* care tailors clinical plans to reduce the effect of social or economic adversity, most often in conventional clinic settings, without necessarily targeting the social condition itself. Social risk-*targeted* care, in contrast, more directly helps patients to reduce social or economic adversity, and is more focused on community intervention. The community-based programs described here fall into both categories in that they seek to provide in vivo services at the individual level focusing on real world adaptation to challenging circumstances while also directly addressing patient-level problems such as housing, criminal justice involvement, impaired activities of daily living, limited employment opportunities, social isolation and a stigmatizing environment. The developing conceptualization of mental health care within a social determinants of health framework, thus provides an overarching context for understanding the unique role of community-focused programs.

Several methodological limitations of this study require comment. First, our ability to identify services delivered through community-based programs is limited to those identified by specific clinic codes in VHA administrative records. It is likely that other programs in VHA that would conform to our concept of community-based care that were implemented through local initiatives, which we could not identify. However, those examined here were developed through national initiatives, often supported by special funding and are probably the largest and best defined.

Second, perhaps the social determinant of health least considered by this study is poverty. The need for income supports is addressed by VA through specific disability compensation and pension programs which provide income benefits for many veterans and which and have been shown to substantially reduce the risk of homelessness [69] . These programs were found, in this study, to be less commonly used by veterans served by outreach to criminal justice involved and by homeless veterans although their access to these benefits has been shown to increase after, and most likely as a result of, participation in VHA’s community-based programs [70]. Crucial data are also not generally available in VA administrative records on the income obtained from social security and local welfare programs. However. specific outreach efforts to facilitate access to social security benefits have been successfully undertaken by VHA in collaboration with the Social Security Administration [71, 72].

Third, the definition of intensive community-based programs is not precise and while most programs addressed here involve frequent contact with veterans outside of health care facilities there is variability from program to program (e.g. criminal justice programs focus on linkage rather than intensive service delivery) and facility to facility in the extent of vivo as contrasted with office-based service delivery in these programs. Nevertheless, all of the programs are intended to address exceptionally serious clinical conditions and specific socially determined challenges to community adaptation.

Fourth, this study focuses on data from the VHA which offers the advantage of providing comprehensive national data from electronic health records. However, VHA is federally funded and operated and serves only veterans, who are overwhelmingly male, and thus its generalizability to other populations and health systems is unknown. The extent to which veterans studied here received non-VA services is also unknown. This study offers a sketch of one system which, it is hoped, will stimulate similar studies of others.

Finally, the data used in this study are somewhat dated as they are now 8 years old. However, 2012 was closer than more contemporary data to the point in time when community-based services emerged in the VHA in response to emerging social needs of several subgroups of veterans. In addition, a recent study [31] found little change in the characteristics of homeless veterans treated by VHA from FY 2008 to FY 2015, a major segment of the population with social challenges that the VHA now serves through community-based programs.

This study could not describe each type of intensive community-based program offered by the VHA in detail but rather, summarized their primary service models and characterize the veterans they serve. We do provide data on the average number of contacts of each program with the veterans it serves.

## Conclusion

In 2012, almost one-fifth of VHA mental health patients received specialized community-based services addressing, most distinctively, major social determinants of health and multimorbid substance use disorders. While the effectiveness, and cost-effectiveness of these individual services has been demonstrated in randomized trials [25, 39, 73, 74], evaluation of the accessibility and effectiveness of such programs in the context of large regional and national service systems is a far more challenging task, and remains to be undertaken.

## Data Availability

The data that support the findings of this study are available from the Veterans Health Administration but restrictions apply to the availability of these data, which were used under license for the current study, and so are not publicly available. Data are however available from the authors upon reasonable request and with permission of the Veterans Health Administration.

## Abbreviations

ACT: Assertive community treatment
FY: Fiscal year
HIV: Human immunodeficiency virus
ICD: International Classification of Diseases
NCS: National Comorbidity Survey
SMI: Severe mental illness
VA: Veterans Affairs
VHA: Veterans Health Administration
